# *Drosophila* MOF controls Checkpoint protein2 and regulates genomic stability during early embryogenesis

**DOI:** 10.1186/1471-2199-14-1

**Published:** 2013-01-24

**Authors:** Sreerangam NCVL Pushpavalli, Arpita Sarkar, M Janaki Ramaiah, Debabani Roy Chowdhury, Utpal Bhadra, Manika Pal-Bhadra

**Affiliations:** 1 Centre for Chemical Biology, Indian Institute of Chemical Technology, Hyderabad, 500607, India; 2 Functional Genomics and Gene Silencing Group, Centre for Cellular and Molecular Biology, Hyderabad, 500007, India

**Keywords:** *Mof*, Mitosis, Syncytial embryos, *Drosophila melanogaster*, *Chk2*, Anaphase bridges

## Abstract

**Background:**

In *Drosophila* embryos, checkpoints maintain genome stability by delaying cell cycle progression that allows time for damage repair or to complete DNA synthesis. *Drosophila* MOF, a member of MYST histone acetyl transferase is an essential component of male X hyperactivation process. Until recently its involvement in G2/M cell cycle arrest and defects in ionizing radiation induced DNA damage pathways was not well established.

**Results:**

*Drosophila* MOF is highly expressed during early embryogenesis. In the present study we show that haplo-insufficiency of maternal MOF leads to spontaneous mitotic defects like mitotic asynchrony, mitotic catastrophe and chromatid bridges in the syncytial embryos. Such abnormal nuclei are eliminated and digested in the yolk tissues by nuclear fall out mechanism. MOF negatively regulates *Drosophila* checkpoint kinase 2 tumor suppressor homologue. In response to DNA damage the checkpoint gene *Chk2* (*Drosophila mnk*) is activated in the *mof* mutants, there by causing centrosomal inactivation suggesting its role in response to genotoxic stress. A drastic decrease in the fall out nuclei in the syncytial embryos derived from *mof*^*1*^*/+; mnk*^*p6*^*/+* females further confirms the role of DNA damage response gene *Chk2* to ensure the removal of abnormal nuclei from the embryonic precursor pool and maintain genome stability. The fact that *mof* mutants undergo DNA damage has been further elucidated by the increased number of single and double stranded DNA breaks.

**Conclusion:**

*mof* mutants exhibited genomic instability as evidenced by the occurance of frequent mitotic bridges in anaphase, asynchronous nuclear divisions, disruption of cytoskeleton, inactivation of centrosomes finally leading to DNA damage. Our findings are consistent to what has been reported earlier in mammals that; reduced levels of MOF resulted in increased genomic instability while total loss resulted in lethality. The study can be further extended using *Drosophila* as model system and carry out the interaction of MOF with the known components of the DNA damage pathway.

## Background

In eukaryotic organisms the individual identity of cells is determined by cell specific genes while a set of genes that are expressed in all cells functions as housekeeping genes. Eukaryotic DNA is highly packaged into chromatin structures, with core histone and non histone chromosomal proteins that regulate many cellular processes including DNA replication and repair of damaged DNA. Regulation of cell cycle involves processes that are crucial to the survival of a cell, wherein detection and repair of genetic damage occurs to control unwanted cell division and maintain genomic stability. Disruption of checkpoint function plays an important role in carcinogenesis and embryonic lethality [1,2]. Chromatin regulatory activities along with histone modifications facilitate the contact of repair proteins at the damaged sites and promote recruitment of components of signaling cascade. Acetylation of lysine16 on histone H4 (H4K16Ac) has the potential to create or obscure binding platforms for chromatin modifying enzymes and transcriptional activators. Furthermore H4K16 acetylation can directly impact on higher order chromatin structure, thus creating an open highly accessible chromatin conformation. The major enzyme that acetylates H4K16 is MOF (Males Absent on First) which is highly conserved in mammals and *Drosophila*.

*Drosophila* histone acetyl transferase MOF is responsible for the interplay between the regulators of transcription and chromatin modifiers thereby governing the gene expression at transcriptional level. It belongs to the family of MYST histone acetyl transferases (HATs) which consists of a conserved catalytic MYST domain [3,4]. The members of this family display diverse roles in various nuclear processes and some of them have also been implicated in carcinogenesis [5]. MOF is an integral member in the *Drosophila melanogaster* dosage compensation process that ensures that males and females, despite unequal number of X chromosomes, express the same amount of X-linked gene products [6]. MOF has strict substrate specificity to H4K16 when compared to other HATs [7,8]. *Drosophila mof* was identified in a screen for ethyl methane sulfonate-induced male-specific lethal mutations and was shown to directly acetylate Histone H4 at K16 [9]. Deletion of *mof* in the case of both *Drosophila* and mammals caused substantial decrease in H4K16 acetylation indicating that *mof* is the major HAT for H4K16 [10,11].

Acetylation of H4K16 by MOF causes reduction in the chromatin compaction *in vitro* and decondensation of chromatin under *in vivo* conditions [12,13]. Hence MOF regulates chromatin based activities such as transcription and DNA damage repair by H4K16 acetylation. Moreover MOF is an important constituent of X-chromosome dosage compensation complex (DCC) resulting in two fold activation of X-linked genes in male flies. Males carrying loss of function *mof* mutation do not survive since they lack the H4K16Ac enrichment on the X-chromosome for transcription of the X-linked genes [6,14]. Interestingly mammalian MOF has high degree of sequence similarity to *Drosophila* MOF protein and H4K16 acetylation is also an epigenetic signature of cellular proliferation during embryogenesis and oncogenesis [15]. Further the role of MOF in ionizing radiation (IR) response is also conserved in *Drosophila*[11]. Recent studies in mammals suggest that the levels of H4K16 acetylation were reduced both in cancer cell lines and primary tumors [16]. Increased genomic instability, with high spontaneous chromosomal aberrations and reduced γ-H2AX foci formation after IR treatment are characteristic features of cultured *mMof*^*+/−*^ cells. In the case of mammals total loss of function (*mof*^*−/−*^*)* resulted in lethality [15].

Faithful transmission of genetic information in cellular organisms is carried out by two basic processes such as DNA replication and cell division. The first 13 syncytial nuclear divisions in *Drosophila* are maternally controlled and consist mainly of S and M phases with short or undetectable gap phases [17]. The syncytial cycles from 1–7 occur inside the embryos and nuclear migration to the cortex occurs during cycles 8 and 9 where further synchronous divisions take place before the onset of cellularisation at 14^th^ nuclear cycle. During cycle 9 few nuclei migrate to the poles to form the pole cells that become the germ cells of the embryo [18]. After completion of 13 syncytial cycles, the embryo undergoes cellularization. Cell cycle checkpoints maintain genomic integrity and stability by regulating the progression of the cell cycle and inducing apoptosis in response to DNA damage to eliminate deleterious mutations from the genome. Defects in cell cycle checkpoints cause a wide variety of defects such as aging, genetic diseases, oncogenesis and neurodegeneration. Proper balance of cell cycle responses are critical for cell death or cell survival to occur. Though DNA damage and replication checkpoint induced apoptosis has been extensively studied, less is known about the cellular responses to stress during mitosis. Checkpoint failures lead to progression of mitosis without damage repair leading to mitotic catastrophe. Embryos exhibiting mitotic catastrophe have giant and fragmented nuclei lacking a regular pattern and 2N ploidy [19].

In the present study we report the identification and phenotypic characterization of *Drosophila* mutants which are haplo-insufficient for maternal MOF. During early embryogenesis mutation in *Drosophila mof* leads to spontaneous chromosomal aberrations and genomic instability leading to mitotic cell cycle progression without repair of damaged DNA. Most significantly, we found the activation of *Drosophila* homolog of the *checkpoint kinase 2* (*DmChk2* or *mnk*) in response to *mof* mutation causing centrosomal inactivation. We propose that *Drosophila* MOF, like its human counterpart, is required for maintaining genomic stability during embryogenesis.

## Results

### *mof* heterozygote embryos are haplo-insufficient for maternal MOF gene product

**Figure 1:**
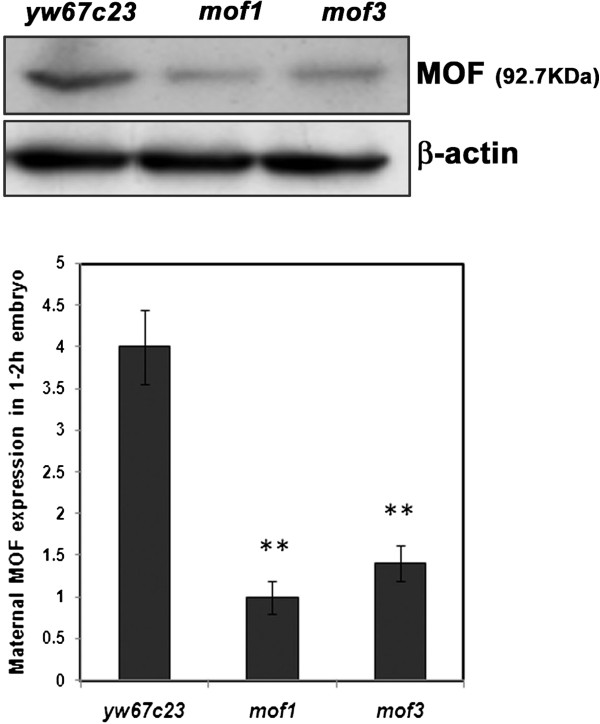
**Haplo-insufficiency of *mof***^***1 ***^**heterozygotes. **Total protein was isolated from early embryos of control (*yw*^*67c23*^*), mof *^*1 *^and *mof *^*3*^ (1–2 h) and western blot analysis carried out using mof antibody has shown 3-fold reduction in the maternal MOF protein. β-actin is used as an internal loading control. Statistical significance was assessed using student t-test. *** indicates P<0.001, ** indicates P<0.01, * indicates p<0.05.

*mof*^*1*^ is a EMS mutation having a single amino acid substitution in the acetyl co-enzyme motif [9]. Sequence analyses revealed that *mof*^*3*^ results from a nonsense mutation at aminoacid 151 (Q151X) [11]. The nature of the *mof* alleles has been studied by quantifying the amount of maternal MOF gene product. For this purpose total protein was isolated from control (*yw*^*67c23*^) *mof*^*1*^ (Ethyl Methane Sulphonate mutation) and *mof*^*3*^ (non-sense mutation) embryos (1–2 h) and western blot analysis was carried out using MOF antibody. A drastic decrease in MOF expression in *mof* heterozygote embryos compared to wild type controls indicated that *mof* mutation is haplo-insufficient for maternal gene product (Figure 1).

### Asynchronous cell cycle and Mitotic catastrophe in the *mof* embryos

**Figure 2:**
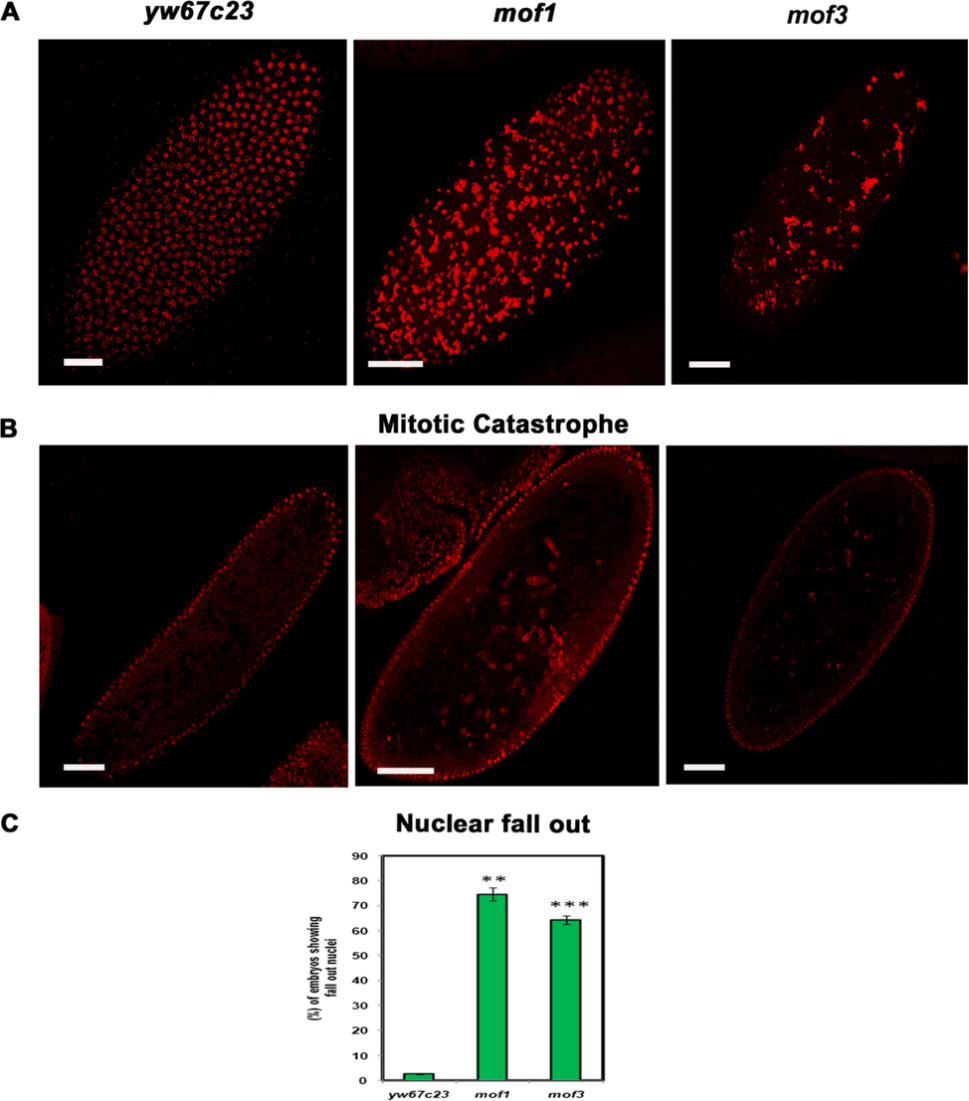
**Loss of MOF causes asynchronous cell cycle, mitotic catastrophe and nuclear fallout. **Early embryos (0–2 h) of *yw*^*67c23*^, *mof*^*1*^ and *mof*^*3*^ were collected, fixed with DNA dye PI and visualized using confocal microscopy. (**A**) Large fragmented nuclei indicating the occurrence of mitotic catastrophe is seen in the early embryos of *mof*^*1*^and *mof*^*3*^ mutants when compared to control embryos. (**B** &**C**) Increased number of fall out nuclei is observed in the *mof*^*1*^ and *mof*^*3*^ mutants when compared to control *yw*^*67c23*^ embryos. Bar indicates 10 μm scale. The data is represented in the form of bar diagram. Statistical significance was assessed using student t-test. *** indicates P<0.001, ** indicates P<0.01, * indicates p<0.05.

MOF is a highly conserved MYST family HAT that acetylates histone at H4K16 and plays an important role in transcriptional activation. Studies of *mof* null mice showed delayed development with massive abnormal chromosomal aggregation, leading to death at an early stage [15]. *In vitro* and as well as *in vivo* studies in *Drosophila* has shown that MOF is required for efficient repair of DNA damage induced by ionizing radiation [11]. Since *mof*^*1*^ mutants are haplo-insufficient for the maternal gene product, we were interested to study the role of MOF during early mitotic divisions that are syncytial. Embryos derived from heterozygous mothers (haplo-insufficiency of maternal gene product) of *mof*^*1*^*/FM7* (EMS mutagenesis), *mof*^*3*^*/FM7* (non-sense mutation) as well as *yw*^*67c23*^ (control) were collected. Early embryos (0–2 h) were fixed and mounted in propidium iodide (PI) to visualise the nuclei. *mof* heterozygote embryos exhibited mitotic catastrophe with fragmented nuclei that appear as large mass of chromatin compared to wild type control where the nuclei appeared normal (Figure 2A). During early embryogenesis the initial seven syncytial divisions occur at the interior of the embryo. During cycles 8 and 9 the nuclei migrate to the cortex leaving only few yolk nuclei. We observed that abnormal nuclei in the *mof* heterozygote embryos are eliminated by nuclear fallout mechanism where in they are digested inside the yolk tissues. Nuclear fallout mechanism protects the organism by eliminating the abnormal nuclei from forming adult structures that might be deleterious. Nearly 70% of the *mof* heterozygotes exhibited a large number of fall out nuclei (high severity fall out nuclei=more than 5 fall out nuclei/embryo) (Figure 2B, 2C) compared to control nuclei (*yw*^*67c23*^) where the number of fall out nuclei is negligible. Hence a decreased number of nuclei are present in the *mof* embryos compared to control (*yw*^*67c23*^). The fall out nuclei in embryos were scored when they are 2-20μm below the cortex in the syncytial blastoderm stage as they are mis-interpretated in later stages where fall out co-exists with normal nuclear migration.

### Abnormal mitosis in *mof* heterozygotes

**Figure 3:**
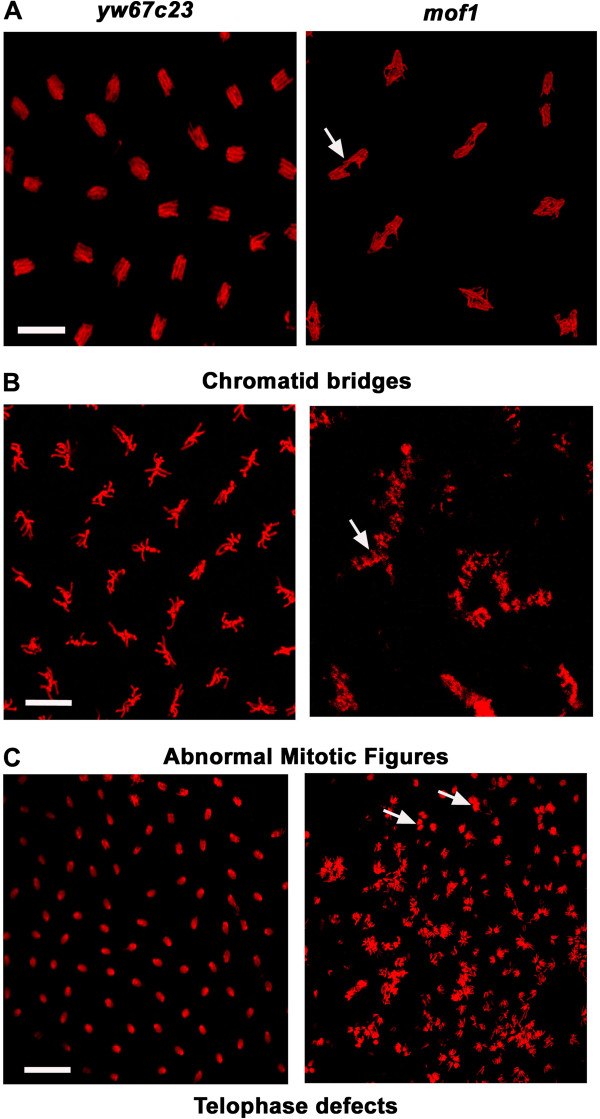
**Loss of maternal MOF causes chromsomal defects in early embryos. **Early embryos (0–2 h) from *mof*^*1*^ and *yw*^*67c23*^ were collected, fixed with DNA dye PI and visualised using confocal microscopy. The *mof*^*1*^ mutants displayed a wide variety of chromosomal defects like (**A**) chromatid bridges which indicates the presence of lagging chromosomes (**B**) sister chromatid separation and (**C**) telophase defects. Bar indicates 10 μm scale.

*Mof* is a maternal effect gene and homozygotes for *mof* mutation do not survive (late larval lethal) till adult stage [11]. To study the role of MOF in early mitosis, we collected embryos derived from heterozygous mothers (haplo-insufficiency of maternal gene product) of *mof*^*1*^*/FM7* and *yw*^*67c23*^ (control). During the early syncytial nuclear divisions, *mof*^*1*^ mutant embryos exhibited several mitotic defects such as chromatid bridges resulting in lagging chromosomes (Figure 3A), defects in sister chromatid separation (Figure 3B); telophase defects (Figure 3C) indicating that *mof* heterozygous embryos may be entering mitosis with damaged or incompletely replicated DNA. The lethality associated with *mof* homozygotes was fully rescued with the addition of *mof* transgene. Although the transgenic line expressing *mof* transgene was viable and fertile, it did not completely restore the chromosomal defects (only 60% of the defects were rescued) (Additional file 1). The embryos from *mof*^*1*^*/+* display similar mitotic defects as that of *mof*^*1*^*/FM7* while the embryos from *FM7/+ f*emales do not show any mitotic defects indicating that *FM7* balancer has no role in causing the mitotic defects observed in the case of *mof*^*1*^*/FM7* embryos (data not shown).

### Mitotic asynchrony during early nuclear divisions in *mof* heterozygous embryos

**Figure 4:**
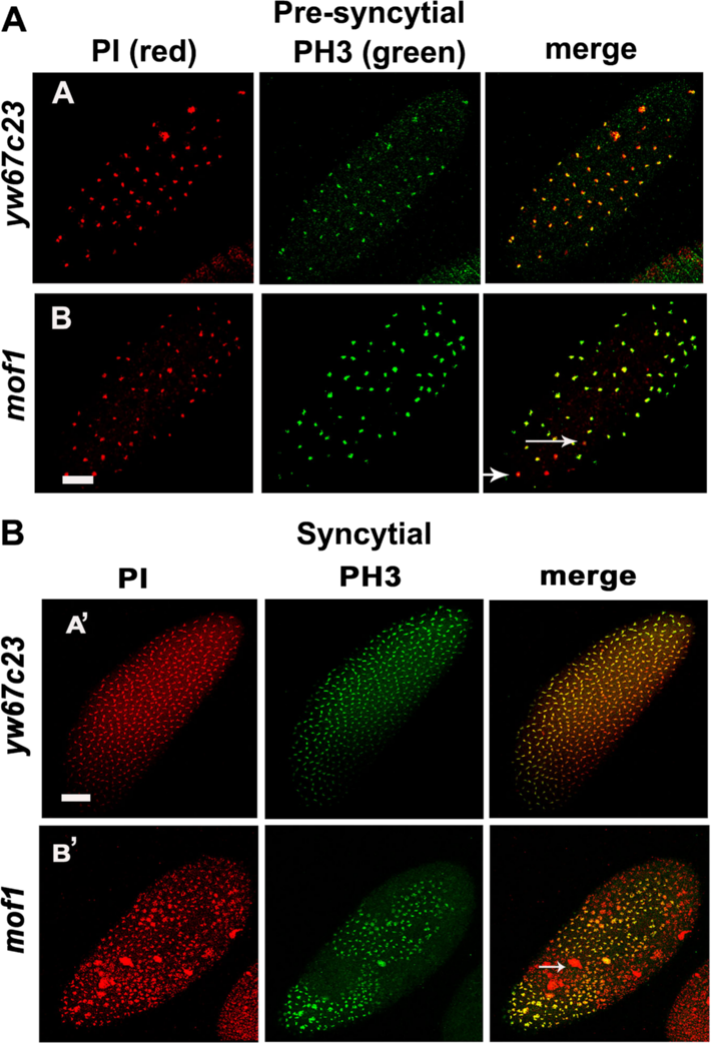
**Mitotic asynchrony in *mof***^***1 ***^**heterozygotes. **Early embryos from 0–2 h were collected from control *yw*^*67c23*^ and *mof*^*1*^ mutants and immunostaining was carried out using PH3 antibody (green) which is mitotic marker. DNA was stained with PI (red). **A** and A’ represents control *yw*^*67c23*^ while **B** and B’ represents *mof*^*1*^ embryos during pre-syncytial and syncytial blastoderm stages respectively. In the case of *mof*^*1*^ embryos the nuclei are unevenly spaced and not all chromsomes are stained with PH3 indicating the existance of mitotic asynchrony during pre-syncytial blastoderm stage. Bar indicates 10 μm scale.

**Figure 5:**
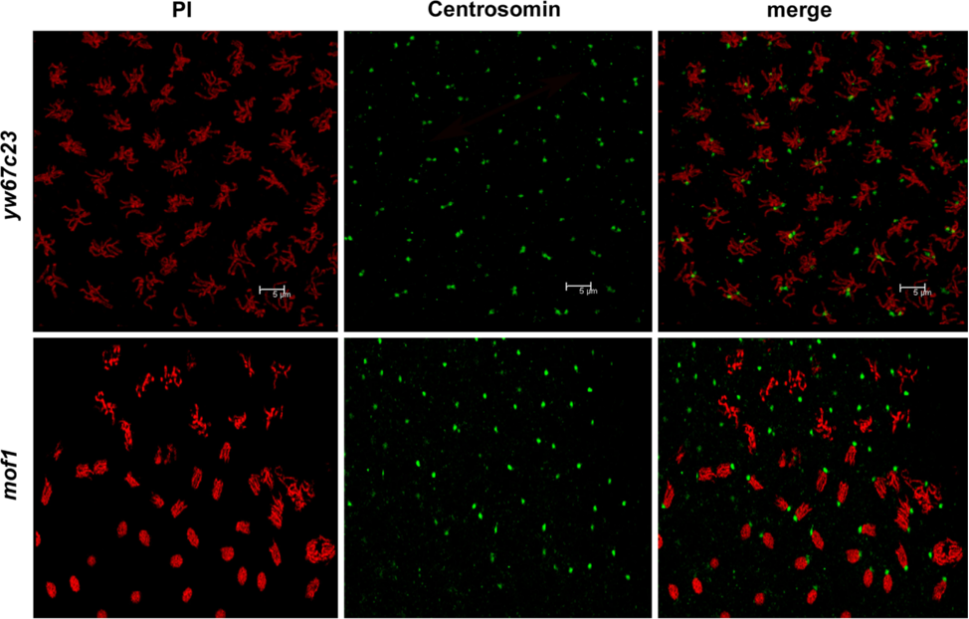
**Loss of MOF results in free centrosomes. **Embryos from 0–2 h from *yw*^*67c23*^ and *mof*^*1*^ mutants were immunostained with anti-centrosomin antibody (green) and DNA was stained using PI (red). Free centrosomes without the chromosomes were present in the *mof*^*1*^ embryos indicating the presence of abnormal nuclei which have been removed by nuclear fallout mechanism. Bar indicates 5 μm scale.

In early embryos of wild type mitosis occurs synchronously and proceeds in the form of waves starting from the poles. Mitotic synchrony during pre-syncytial and syncytial divisons in *mof*^*1*^ and control embryos was studied by staining with antibody against Histone H3 Ser10 Phosphorylation (PH3) (the mitosis marker). Control embryos showed PH3 staining on all the chromsomes while in the case of *mof*^*1*^heterozygotes both PH3 positive (dividing) and PH3 negative (non-dividing) chromosomes were observed. Thus the PH3 negative chromosomes in *mof*^*1*^ heterozygotes indicate the existence of abnormal nuclei. Our data indicates that maternal supply of MOF is required for mitotic synchrony in pre-syncytial and syncytial blastoderm embryos (Figure 4A-A’, B-B’). These abnormal nuclei which loose association with cortex (fall out nuclei) are unlikely to divide since they do not have centrosomes attached to them. To further confirm nuclear fallout early embryos of *mof*^*1*^ heterozygotes and *yw*^*67c23*^ were immunostained with anti-centrosomin antibody*.* A number of free centrosomes lacking the chromosomes were present in the *mof*^*1*^ heterozygous early embryos compared to control embryos (*yw*^*67c23*^). The free centrosomes in the embryo indicated the presence of abnormal nuclei that are eliminated by the nuclear fallout mechanism (Figure 5). In addition to free centrosomes we also observed chromosomes lacking centrosomes or with only one centrosome. These findings strongly suggest the involvement of centrosome inactivation in the *mof*^*1*^ early embryos.

### Disruption of cytoskeleton in the *mof* heterozygous embryos

**Figure 6:**
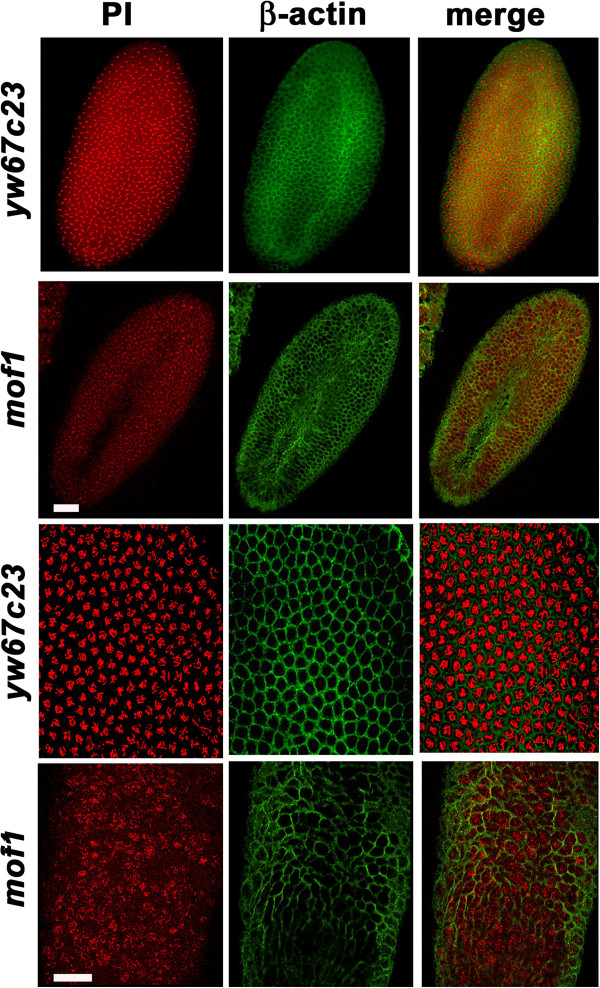
**Defects in actin cytoskeleton in the *mof***^***1 ***^**heterozygotes. **Early embryos (0–2 h) were stained with β-actin (green) antibody in both *yw*^*67c23*^ and *mof*^*1*^ embryos*.* PI is used to stain the DNA. The honeycomb like structure which is characteristic of actin cytoskeleton is largely disrupted by the *mof* mutation. There are few cells which do not have chromsomes in them indicating abnormal nuclei which have been dropped into the cortex and digested by the yolk nuclei. Bar indicates 10 μm scale.

**Figure 7:**
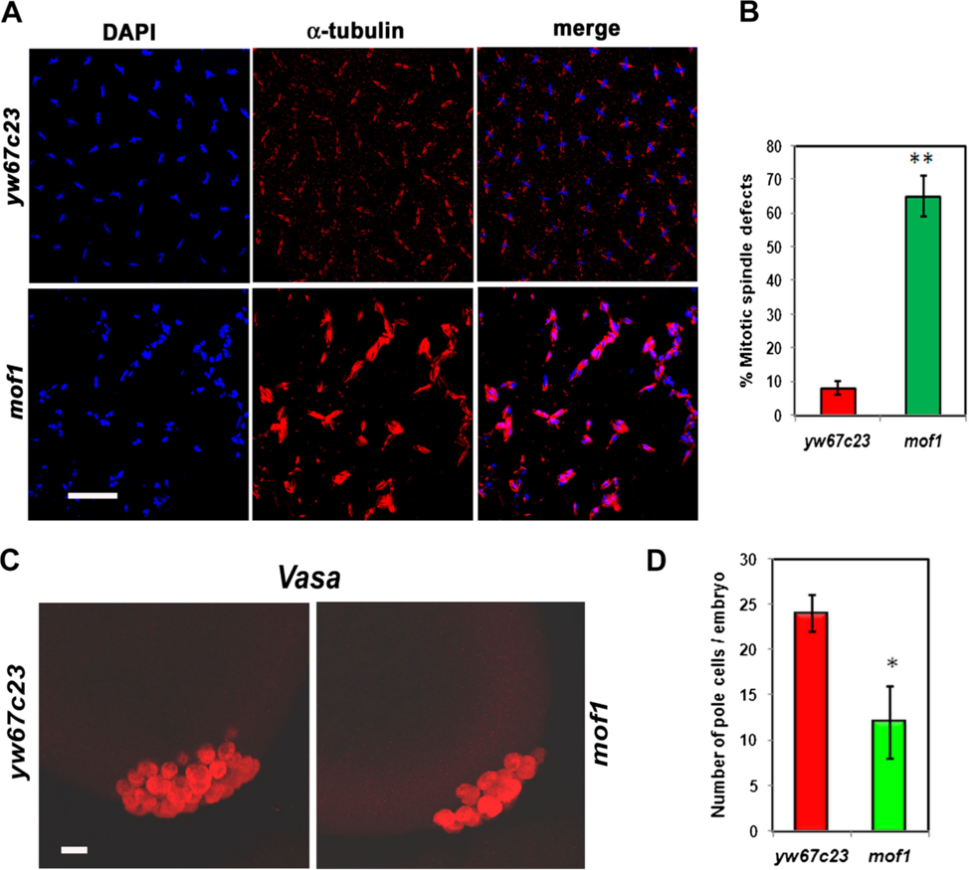
**Loss of MOF causes defects in spindle fibre organisation and nuclear migration. **(**A**) 0–2 h *mof*^*1*^ and *yw*^*67c23*^ embryos were stained with alpha tubulin antibody (red) and DAPI is used as DNA dye. The chromosomes were not properly aligned in the metaphase plane with tubulin fibres attached all over the chromosmes. (**B**) The percentage of embryos exhibiting mitotic spindle defects is represented in the form of bar diagram. (**C**) The early embryos of *mof*^*1*^ and *yw*^*67c23*^ were stained with vasa antibody to visualise the pole cells. There was drastic decrease in the number of pole cells in the *mof*^*1*^ embryos indicating that nuclear migration is affected. (**D**) The number of pole cells in control and *mof*^*1*^ mutant per embryo is represented in the form of bar diagram. Bar indicates 10 μm scale. Statistical significance was assessed using student t-test. *** indicates P<0.001, ** indicates P<0.01, * indicates p<0.05.

Cytoplasmic organization, nuclear division and nuclear migration in the syncytial embryos are modulated by the cytoskeletal proteins. Following the syncytial divisions individual cells are produced by a process called cellularization that occurs during interphase of nuclear cycle 14. Thus we were interested to study the changes in the organization of actin cytoskeleton and hence control *yw*^*67c23*^ and *mof*^*1*^ heterozygous embryos were immunostained with ß-actin antibody. The typical honeycomb like structure of actin cytoskeleton observed in the control was lacking in the case of *mof*^*1*^ embryos. Moreover in the *mof*^*1*^ embryos chromosomes were incompletely surrounded by the actin filaments along with few small cells that lack nuclei. These empty cells indicate the presence of abnormal nuclei which have been eliminated by nuclear fall out mechanism (Figure 6). In addition to the actin filaments the polymerization and depolymerization of microtubule network helps in mediating the coordinated nuclear movement (chromosomes) during syncytial stage of embryogenesis. Since polymerization and depolymerization of the microtubules is required for proper chromosome movement, we stained the *yw*^*67c23*^ and *mof*^*1*^ embryos with alpha-tubulin antibody to visualize the organization of spindle fibres. Around 66% of *mof*^*1*^ embryos as opposed to only 7% of *yw*^*67c23*^ embryos exhibited attachment of spindle fibres all over the chromsomes instead of the kinetochore, indicating disruption of the spindle fibre assembly and therefore leading to improper movement of chromosomes during anaphase resulting in lagging chromosomes. Number of embryos counted in the present study is 100 (Figure 7A, 7B).

The integrity of cell’s cytoskeleton is crucial for the first occasion of vasa localization in the preplasmic cytoplasm as well as second occasion in the pole plasm. Proper function of the cytoskeleton is important for nuclear migration leading to formation of pole cells. Here *yw*^*67c23*^ and *mof*^*1*^ embryos were immunostained with antibody against vasa which selectively stains the pole cells. As anticipated we observed drastic reduction in the number of pole cells in the *mof*^*1*^ embryos compared to control (*yw*^*67C23*^) indicating that MOF is required for proper nuclear migration and formation of pole cells (Figure 7C, 7D).

### Elevated levels of DNA damage in *mof* heterozygous embryos

**Figure 8:**
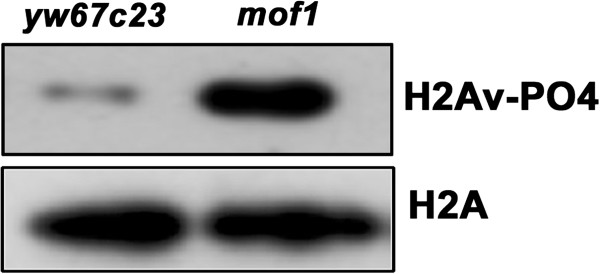
***mof***^***1 ***^**mutation causes double strand breaks in DNA. **Total protein was isolated from *yw67c23* and *mof1* heterozygous embryos and western blot was carried out using H2Av-PO4 antibody. Here H2A antibody was used as loading control. An increase in the level of phosphorylation of H2Av was observed in the *mof1* heterozygotes.

We next wanted to study more specific role of MOF in DNA damage. Thus *mof*^*1*^ heterozygote embryos as well as control embryos were used in an assay that determines the extent of DNA damage (single, double stranded DNA breaks) [20]. Genomic DNA was isolated from the embryos (0–2 h) and incubated with T4 DNA kinase and 32P ATP. The amount of incorporation of 32PATP determined the number of exposed 5’ phosphate groups in the DNA, indicating the number of single and double stranded lesions. Genomic DNA from *mof*^*1*^ embryos showed a highly elevated level of P32 incorporation of 6500 ± 370 cpm/ng compared to the controls which is 1011 ± 200 cpm/ng suggesting that the increase in lesions in *mof*^*1*^ embryos is due to progression through mitosis with damaged DNA or incompletely replicated DNA. To further confirm the double strand breaks observed in the *mof*^1^ heterozygote we carried out western blot studies using phospho H2Av antibody. As expected in *mof*^*1*^ heterozygotes we observed an increase in the levels of H2Av-phosphorylation when compared to control (Figure 8).

### Abnormal nuclei are eliminated by Chk2 activation

Late syncytial embryos of *Drosophila* exhibit two-stage response to DNA damage or replication defects [21]. Two different kinase pathways, ATM/Chk2 pathway and ATR/Chk1 pathway play a major role in response to DNA damage that is evolutionarily conserved. The DNA checkpoint mediated by *mei-41* and *grp*, the *Drosophila* orthologs of *ATR* and *Chk1* kinases, respectively, delay entry into mitosis via inhibitory phosphorylation of Cdk1, which allows repair of DNA damage or completion of DNA replication [22,23]. When this checkpoint fails, a second control operating during mitosis is activated, that results in changes in spindle structure and chromsome segregation to stop propagation of defective or damaged nuclei. This second step of control is mediated by activation of *Chk2* by centrosomal inactivation [24]. The increased number of fall out nuclei and defects during early mitosis in the *mof* heterozygous embryos led us to speculate the possible involvement of a DNA replication dependent or DNA damage dependent cell cycle checkpoint defect. *Drosophila* embryos that lack *Chk1* homologue (*grp)* and *ATR* homologue (*mei-41*) show inactivation of centrosome during the late stages of syncytial division proving that both the homologues are not required for centrosome inactivation [21].

**Figure 9:**
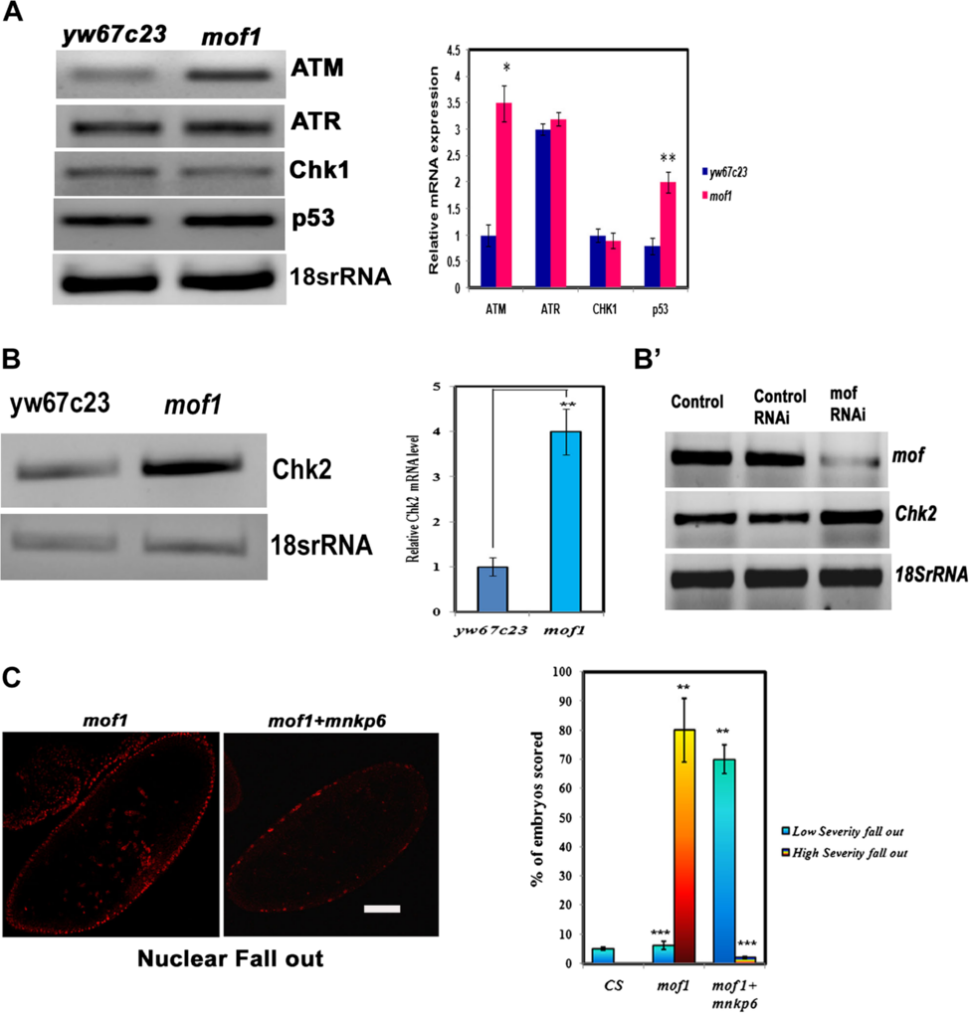
**Activation of *Chk2 *in the *mof *heterozygous embryos. **(**A**) Total RNA was isolated from embryos of syncytial cycles of 10–13 from control *yw*^*67c23*^ and *mof*^*1*^ embyros and semi-quantitative RT-PCR was carried out using primers against *ATM, ATR, Chk1* and *p53* to study their expression pattern. (**B**) Semi-quantitative RT-PCR was carried out using *chk2* primers in *yw*^*67c23*^ and *mof*^*1*^ embryos (syncytial cycles 10–13). We observed 4-fold increase in the level of *chk2* in *mof*^*1*^ embryos compared to control indicating activation of *chk2*. (**B**’) Depletion of MOF by RNAi was performed in S2 cells (*Drosophila* Schneider cells) by incubation with dsRNA against *mof*. The transfection was conducted for 72 h time period. Total RNA isolated was subjected to RT-PCR analysis against *Chk2* and *mof*. Here dsRNA against GFP was used as control (control RNAi). The depletion of mof leads to an increase in the levels of *Chk2* mRNA. (**C**) Early embryos from *mof*^*1*^ and *mof*^*1*^*/+; mnk*^*p6*^*/+* females were collected and stained with DNA dye PI to study the severity of nuclear fallout. The high severity of nuclear fallout in *mof*^*1*^ embryos was drastically reduced in the presence of *mnk*^*p6 *^*(chk2)* mutation and is graphically represented. Statistical significance was assessed using student t-test. *** indicates P<0.001, ** indicates P<0.01, * indicates p<0.05. Bar indicates 10 μm scale.

To show that DNA replication checkpoint is intact in the *mof*^*1*^ embryos, we studied the levels of *grp (Chk1)* and *mei-41* (*ATR*) and we found that their levels remained the same in syncytial cycles 10–13 (Figure 9A). Also the levels of cyclins remained the same in the wild type and *mof*^*1*^ embryos in syncytial cycles 10–13 indicating that our data do not support a role of *Drosophila mof* in the control of cell cycle in the syncytial embryos through regulation of cyclins and *grp*. Thus our data do not support a role for *Drosophila* MOF in control of cell cycle timing in syncytial embryos via regulation of cyclins or *grp* levels.

The defective mitotic spindles that are short, anastral and associated with poorly aligned chromosomes in the *mof* embryos exhibited key features reminiscent of *Chk2* mediated centrosomal inactivation. This led us to investigate the possible role of checkpoint gene *Chk2* in this event. Total RNA was isolated from syncytial cycles 10–13 of control *yw*^*67c23*^*, mof*^*1*^ heterozygotes and RT-PCR was carried out using *Chk2* specific primers. We observed increase in the transcript level of *Chk2* by 4-folds in the *mof*^*1*^ embryos compared to *yw*^*67c23*^ control indicating *Chk2* mediated centrosome inactivation (Figure 9B). To further confirm MOF mediated *Chk2* regulation we have conducted the *mof* knock down experiment using dsRNA in S2 cells. The expression of *Chk2* was found to be enhanced upon *mof* depletion. *GFP* dsRNA did not cause any significant change in levels of mof and thus used as control in the RNAi study (Figure 9B’).

Since *Chk2* is a major target of ataxia telangiectasia-mutated (ATM), the expression pattern of ATM in *mof*^*1*^ heterozygous embryos was also studied. We observed that there was pronounced increase in levels of ATM in *mof*^*1*^ heterozygotes. As reported earlier [11] we also observed increased expression of *p53* in *mof*^*1*^ heterozygous embryos indicating that *mof* mutation causes spontaneous DNA damage leading to the activation of ATM-Chk2 pathway (Figure 9A).

### Centrosome inactivation in asynchronous nuclei of syncytial *mof* heterozygous embryos

*Drosophila Chk2* is encoded by *mnk* (*maternal nuclear kinase*) gene [25] and *mnkp6* homozygous null mutation flies produce DNA damage induced apoptosis [26]. To further confirm *ATM/Chk2* mediated centrosomal inactivation, we crossed *mof*^*1*^*/FM7* virgin females with *mnkp6* males to produce heterozygous *mof*^*1*^*/+; mnk*^*p6*^*/+* flies*.* The *mof*^*1*^*/+; mnk*^*p6*^*/+* females were further mated with wild type males and 0–2 h embryos were collected. These embryos were stained with PI to check for nuclear fallout. The *mof*^*1*^ embryos exhibited high severity fall out nuclei (more than 5 fall out nuclei/embryo) while *mof*^*1*^*/+; mnk*^*p6*^*/+* embryos had only low severity fall out nuclei (less than 5) (Figure 9C). Thus Chk2 activation contributes significantly to the *mof*^*1*^ phenotype in syncytial embryos.

## Discussion

MOF is a member of MYST family of histone acetyl transferases and is the essential component of the X-chromosome dosage compensation system in *Drosophila*. All MOF deficient mouse embryos fail to develop the expanded blastocyst stage and die at implantation *in vivo*. In the present study we observed loss of maternal MOF in the *Drosophila* embryos caused mitotic defects during early syncytial cycles and chromosomal aberrations as visualized by the presence of chromatid bridges and lagging chromosomes. These defects occur spontaneously in *mof* heterozygyotes without stress and resemble the defects induced by X-ray irradiation or chemical treatment of wild type embryos [27]. Our data has shown that endogenous DNA damage occurs during the process of development as shown by the presence of single and double stranded DNA breaks. Similar phenotypes like spontaneous mitotic defects and chromosomal aberrations in *Drosophila* were also observed in *recq5* DNA helicase mutants that are involved in DNA replication and maintainance of genomic integrity [28]. MOF is an essential component of dosage compensation complex and homozygotes for the mutation do not survive beyond late larval stages. The nuclei in *mof* early embryos are large and fragmented resembling mitotic catastrophe. The typical mitotic wave in wild type embryos is disrupted due to the presence of abnormal nuclei in the *mof* heterozygotes. Such abnormal nuclei are PH3 negative and appear in clusters [29]. Cellularisation occurs during the interphase of 14^th^ nuclear division. During this stage the actin filaments in *mof*^*1*^ embryos loose the typical honeycomb like structures often leading to empty cages without the chromosomes. The empty cages are indicative of the presence of abnormal nuclei that are PH3 negative and have been eliminated by nuclear fallout while the centrosomes are still retained in the cortex. A small percentage of embryos also show telophase defects like rounding off of the nuclei while the chromosomes are still in the anaphase stage. Similar mitotic defects were also observed in the case of another *mof* allele (*mof*^*3*^). The late larval lethality of *mof* homozygotes were rescued to 100% with the addition of *mof* transgene in the mutant genetic background while the mitotic defects in the *mof* heterozygote embryos were partially rescued.

In general every organism tries to protect itself by preventing these abnormal nuclei from being incorporated into forming adult structures [30]. Mutation of PcG genes which are components of chromatin remodeling that aid in the maintenance of transcriptional state during embryogenesis also resulted in the formation of abnormal nuclei [19]. Also the severity of the fall out nuclei in the *mof*^*1*^ heterozygotes was high containing more than 5 fall out nuclei per embryo which was reverted back to normal in the presence of wild type *mof* transgene. The response to DNA damage and mitotic defects maintain genomic stability by blocking chromosome segregation and removing the abnormal nuclei by nuclear fallout mechanism. Staining with PH3 antibody and DNA dye in the syncytial embryos is a good system to detect irregular or damaged DNA in *Drosophila* and also for studying maternal genes required for mitosis and genomic stability. The abnormal nuclei that stain negatively for PH3 are asynchronous and are seen during nuclear cycles 11–13. Following mitotic failure the defective nuclei drop into the interior of embryos and free centrosomes are seen in the cortex [29]. In the wild type embryos all the nuclei are in actively dividing phase compared to *mof* heterozygote where in non-dividing nuclei are also present.

In a variety of systems, cell cycle checkpoint defects lead to progression into mitosis with damaged DNA or incompletely replicated DNA leading to “mitotic catastrophe”, a process that is distinct from apoptosis [31]. In syncytial *Drosophila* embryos damaged or incompletely replicated DNA triggers centrosome inactivation during mitosis leading to defects in spindle fibre assembly and chromosome segregation [24]. The hallmark of DNA damage response (DDR) involves the phosphorylation of histone variant H2AX that play an essential role in the recruitment and retention of downstream proteins involved in DNA repair. In addition to this γ–H2AX is also involved in the transduction and amplification of DDR from megabase domains surrounding the damage site [32]. In our studies using *mof*^*1*^ heterozygotes we observed increase in single and double stranded DNA breaks and as well as H2Av phosphorylation revealing the occurrence of DNA damage event.

*Drosophila* Chk2 plays a vital role in response to stress. DNA damage leads to increased localization of Chk2 to centrosomes and spindle fibres and also Chk2 is the signal for mitotic catastrophe that disrupts centrosome function leading to elimination of the abnormal nuclei [24]. It was also reported that mutation of *Mnk* gene (*Drosophila* homolog of *Chk2*) prevents centrosome inactivation and suppresses defects associated with chromosome segregation in response to damaged or incompletely replicated DNA. In our study we observed increased level of Chk2 in the *mof* heterozygotes in response to the damaged nuclei causing centrosome inactivation resulting in elimination of the damaged nuclei. The number of abnormal or damaged nuclei was reduced in the embryos of *mof*^*1*^*/+; mnk*^*p6*^/+ mothers indicating that mutation of *mnk*^*p6*^ prevents inactivation of centrosomes and hence loss of nuclei from the cortex. Unlike the cell cycle delays that occur to repair the damaged DNA or incompletely replicated DNA, *Drosophila* embryonic system utilizes the delay to identify and discard those abnormal nuclei. When the DNA lesions enter into mitosis, Chk2 is activated as a response and leads to centrosomal inactivation and delinks the chromosomes from their centrosomes which ultimately results in loss of the nuclei [29]. It has been proposed that Chk2 functions at two points during early embryogenesis in response to genotoxic stress. At the onset of mitosis DNA lesions leads to activation of Chk2 that target proteins involved in centrosomal spindle activity and in maintaining γTURC localisation. This causes failure in anaphase chromosome segregation. Once failure of mitotic division occurs, Chk2 causes centrosomal inactivation and disrupts the link between centrosomes and nuclei. Since centrosomes anchor nuclei to the cortex, Chk2 response to DNA damage results in loss of nuclei from the cortex [33]. The sensing of DNA lesions by DDR machinery occurs in a complex and heterogeneous chromatin environment [34,35]. Earlier reports also emphasized on the alteration in the chromatin structure that helps in the sensing and as well as spreading of the DNA damage response apart from double strand breaks [36-38].

DNA damage induces the activation of chromatin bound Chk2 by a chromatin derived signal resulting in the dissociation of the activated Chk2 from the chromatin. Chk2 is phosphorylated at T68 by ataxia telangiectasia-mutated (ATM) and transmits the DNA damage signals from the upstream phosphatidylinositol 3’-kinase like kinases to the effector substrates including p53, Braca1, Cdc25A and Cdc25C [39]. In addition Chk2 has been reported to phosphorylate p53, thereby enhancing the transcriptional activity of p53 responsive genes [40]. Further the functional link between p53 and Chk2 during DNA damage occurs through the phosphorylation and acceleration of degradation of Hdmx, a negative regulator of p53 [41].

This study revealed for the first time the role of MOF during early embryogenesis in *Drosophila* apart from dosage compensation and response to ionizing radiation*.*

## Conclusions

Recent investigations have clearly demonstrated the role of MOF in response to ionizing radiation is conserved in *Drosophila melanogaster*. In human cells knockdown of hMOF results in loss of H4K16Ac and destabilization of nucleosomes that correlates with regions of chromatin decondensation. While acetylated H4 K16 appears to ‘open up’ the *Drosophila* male X chromosome to make it more accessible to transcription, which is an important part of the dosage compensation mechanism in the fly. Reduced levels of MOF in mammals correlate with decreased H4K16Ac, cell proliferation, cell survival and increased genomic instability. *Drosophila* haplo-insufficency of maternal MOF causes several mitotic defects in the syncytial embryos and a large number of abnormal nuclei have been removed through the process of nuclear fallout. The increased number of abnormal or fall out nuclei correlated with reduced nuclear density in syncytial blastoderm embryos of *mof* heterozygotes. Our study demonstrates that in response to spontaneous DNA damage (increased number of single and double stranded DNA breaks) in *mof* heterozygotes, Chk2 is activated leading to centrosomal inactivation and loss of damaged nuclei from the cortex of the syncytial embryos. Furthermore removal of one copy of *Chk2* in the *mof* mutant background considerably reduced the number of fall out nuclei in the syncytial embryos indicating the restoration of genomic stability. Hence MOF seems to play a crucial role in ensuring genomic stability during early embryogenesis both in mammals and *Drosophila.*

## Methods

### Fly stocks

Flies were cultured at standard corn meal agar food at 25°C. *yw*^*67c23*^ was obtained from flybase. *mof*^*1*^*, mof*^*3*^, *mof*^*3*^*+18H1*and *mnk*^*p6*^ fly stocks were kind gift from John C. Lucchesi, Joel C. Eissenberg and William E. Theurkauf respectively. Both *mof*^*1*^ and *mof*^*3*^ are null alleles of *mof*[11].

### Immunostaining of *Drosophila* embryos

*Drosophila* embryos were dechorionised with 50% commercial bleach for 2–3 minutes and fixed with paraformaldehyde/heptane mix for 15–20 min. Primary antibodies for immunostaining were used in the following dilution: goat anti-vasa (1:20), rat anti-α-tubulin (1:20), rabbit anti-PH3 (1:50), mouse anti-β-actin (1:30). Goat or donkey raised FITC, Cy3 and Cy5 conjugated secondary antibodies were used at a dilution of 1:50. Embryos were then mounted in Vectashield mounting media containing PI or DAPI and viewed using confocal microscope (FV1000, Olympus, Japan).

### Total RNA isolation and RT- PCR analysis

Total cellular RNA from embryos was isolated by Trizol (Invitrogen). RNase-Free DNase treatment was subsequently carried out to remove DNA contaminants and further cleaned using RNeasy Mini Kit (Qiagen, Germany). Three micrograms of RNA was used for first strand cDNA synthesis using SuperScript™ (Invitrogen, USA). PCR analysis was carried out using the following primers

18SrRNA

FP- 5’ CCTTATGGGACGTGTGCTTT 3’,

RP- 5’ CCTGCTGCCTTCCTTAGATG 3’

Chk2

FP-5’ CAAGCTGCTGATCAACCAAA 3’

RP-5’ GCCTCGACCCTCACGTATTA 3’

ATM

FP-5’ ATCATAGCTTGGGCATACGG3’

RP-5’ TTTGTTCTCCTTCGCGATCT3’

p53

FP-5’AAT GCC CAT CCA ACC ACT TA3’,

RP-5’AAG GCT CAA CGC TAA GGT GA3’

Mof

FP-5’CTGGGTAGGCTGAGCTATCG3’,

RP-5’ CCAGACGAGGTAATCGGTGT3’

Chk1/grp

FP-5’CCGGACTCAATTACCTGCAT3’,

RP-5’GTTTGCTCCAAGGAGTCTGC 3’

ATR/Mei-41

FP-5’ TCAGGAGACGCTAGCCATTT3’

RP-5’ TGCAGAACTGCCATGAACTC 3’

### Total protein isolation and western blot analysis

Nearly 0.1 gm of embryos were collected and thoroughly homogenized in lysis buffer (6% SDS, 1 mM EDTA, 2 mM PMSF, 10 μg/ml Aprotinin, 10 μg/ml Leupeptin, 10 μg/ml Pepstatin). The lysed samples were boiled at 95°C for 5 minutes and centrifuged at 12,000 rpm for 10 minutes at 4°C. The supernatant was collected and western blot analysis was carried out by standard protocols using MOF (1:300), ß-actin (1:500) (Abcam) and H2Av-PO4 (1:500) antibodies (LS biosciences).

### DNA damage assay

In order to assess DNA damage, genomic DNA was isolated from 0–2 h methanol-fixed wild type control and *mof*^*1*^ embryos as described earlier with slight modifications [21]. NaOH lysis is followed by incubation at 65°C for 40 min. The embryos were gently homogenised and treated with proteinase K for 8 h at 55°C, followed by RNAse A treatment and phenol-chloroform extraction. Each sample of DNA (100 ng) was incubated at 37°C for 20 min in 25 μl of a reaction mixture containing 50 mM imidazole pH 6.4, 12 mM MgCl2, 1 mM β-mercaptoethanol, 100 μM ADP, 20 units T4 DNA Kinase (Gibco BRL) and 2.5 μl of 3000 Ci / mmol [32P]ATP (Amersham). Column purification was carried out using G-50 spin columns to remove unincorporated labeled nucleotides. The level of p32 incorporated into the genomic DNA was measured by liquid scintillation counter. Over 300 embryos were used to generate the DNA for each experiment and the experiments were repeated thrice.

### Knock down studies

MOF knockdown by RNAi was conducted in S2 cells using Schnieder’s insect medium supplemented with 10% fetal bovine serum at 25°C. Here for the effective knock down of *mof,* we transfected 50 μg dsRNA in S2 cells and were incubated for 72 h as described earlier [11]. Control RNAi experiments using GFP was also employed.

### Statistical analysis

Statistical analysis was performed using the graph pad software to evaluate the significant difference between the control and treated samples. The results obtained were expressed as mean ± SD. All the experiments were conducted in triplicates. Statistical significance was assessed using student t-test. *** indicates P<0.001, ** indicates P<0.01, * indicates p<0.05.

## Abbreviations

Mof: Males absent on the First; HAT: Histone acetyl transferase; DCC: Dosage compensation complex; PI: Propidium Iodide; DDR: DNA damage response; mnk: Maternal nuclear kinase; PH3: Anti-phospho-Histone H3 (Ser10).

## Competing interests

The authors declare that they have no competing interests.

## Authors’ contributions

SNCVLP, AS, MJR and DRC carried out the experiments. SNCLVP and MJR designed the experiments. UB and MPB conceived the idea, designed the experiment and wrote the manuscript. All authors read and approved the final manuscript.

## Supplementary Material

Additional file 1 **Rescue of mitotic defects in *mof *embryos by *mof *transgene.** Click here for file
