# Supplementary material for: Drosophila MOF controls Checkpoint protein2 and regulates genomic stability during early embryogenesis
Source: BMC Mol Biol. 2013 Jan 24;14:1. doi: 10.1186/1471-2199-14-1 (PMC3566930; doi:10.1186/1471-2199-14-1)

**Supplementary**

**Figure S1. Rescue of mitotic defects in *mof* embryos by *mof* transgene**

(A) Early embryos from females of *mof3+ mof* transgene were collected, processed and stained with DNA dye PI. The mitotic defects were rescued by the *mof* transgene and the embryos appear normal with (A) mitotic synchrony and no mitotic catastrophe (B) without nuclear fall out (C) sister chromatid separation and (D) chromatid bridges.

(B) *mof3* mutant females were crossed with *mof* transgene males and survival of genotype *mof3+mof* transgene were assayed. The resulting data was quantified and represented as bar diagram. Addition of *mof* transgene in the background of *mof3* mutant could completely rescue the lethality associated with the *mof* mutation.

(C) *mof3* mutant females were crossed with *mof* transgene males. The resulting embryos from mothers of *mof3 +mof* transgene were studied for mitotic defects and the data is represented in the form of histogram. The mitotic defects in the *mof3* mutants were rescued upto 60% with the addition of *mof* transgene.

**A**


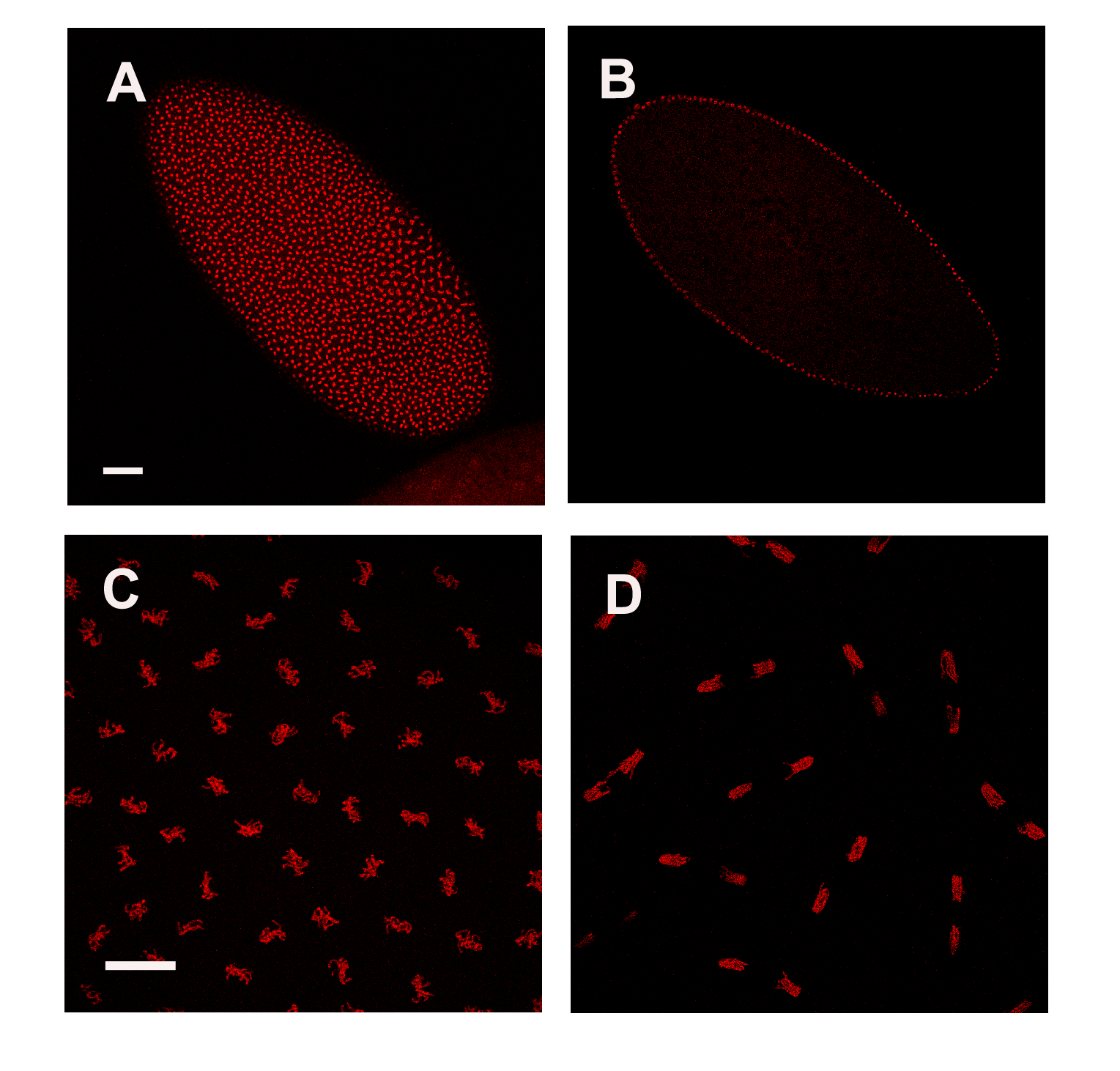


**B**


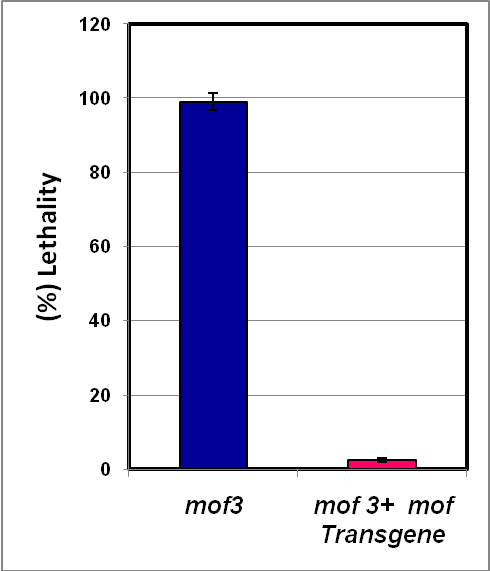


**C**


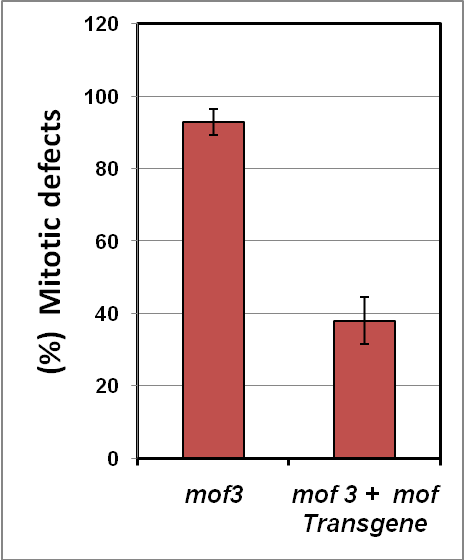

Supplement: Additional file 1 — Rescue of mitotic defects in mof embryos by mof transgene. [file 1471-2199-14-1-S1.doc]
